# A Modified Janus Cassette (Sweet Janus) to Improve Allelic Replacement Efficiency by High-Stringency Negative Selection in *Streptococcus pneumoniae*

**DOI:** 10.1371/journal.pone.0100510

**Published:** 2014-06-24

**Authors:** Yuan Li, Claudette M. Thompson, Marc Lipsitch

**Affiliations:** Department of Epidemiology and Department of Immunology & Infectious Diseases, Harvard School of Public Health, Boston, Massachusetts, United States of America; Centers for Disease Control & Prevention, United States of America

## Abstract

The Janus cassette permits marker-free allelic replacement or knockout in streptomycin-resistant *Streptococcus pneumoniae* (pneumococcus) through sequential positive and negative selection. Spontaneous revertants of Janus can lead to high level of false-positives during negative selection, which necessitate a time-consuming post-selection screening process. We hypothesized that an additional counter-selectable marker in Janus would decrease the revertant frequency and reduce false-positives, since simultaneous reversion of both counter-selectable makers is much less likely. Here we report a modified cassette, Sweet Janus (SJ), in which the *sacB* gene from *Bacillus subtilis* conferring sucrose sensitivity is added to Janus. By using streptomycin and sucrose simultaneously as selective agents, the frequency of SJ double revertants was about 10^5^-fold lower than the frequency of Janus revertants. Accordingly, the frequency of false-positives in the SJ-mediated negative selection was about 100-fold lower than what was seen for Janus. Thus, SJ enhances negative selection stringency and can accelerate allelic replacement in pneumococcus, especially when transformation frequency is low due to strain background or suboptimal transformation conditions. Results also suggested the *sacB* gene alone can function as a counter-selectable marker in the Gram-positive pneumococcus, which will have the advantage of not requiring a streptomycin-resistant strain for allelic replacement.

## Introduction

Genetic manipulations of *Streptococcus pneumoniae* (pneumococcus) have been a powerful approach to understand virulence factors and drug targets in this important human pathogen. In particular, the Janus cassette [1] permits marker-free allelic replacement or knockout in *Streptococcus pneumoniae* through sequential positive and negative selection [2]–[4]. The Janus comprises a kanamycin-resistance (Km^r^) marker and a counter-selectable *rpsL* ^+^ marker that confers dominant streptomycin-sensitivity (Sm^s^) in a streptomycin-resistant (Sm^r^) background [1]. Allelic replacement is accomplished in a Sm^r^ parental strain by a 2-step transformation procedure. The first transformation tags the locus of interest with the Janus cassette by homologous recombination while conferring a Km^r^ phenotype that can be selected (positive selection) and Sm^s^. The resulting Janus-carrying strain (Km^r^Sm^s^) is then transformed by donor DNA to replace Janus with an allele of interest. The loss of Janus and acquisition of the allele of interest after the second transformation result in a Sm^r^ phenotype that can be selected (negative selection) and Km^s^. However, Sm^r^ can also result from spontaneous conversion of the *rpsL* ^+^ marker in Janus into a Sm^r^ *rpsL* allele (spontaneous revertants), which occurs at a frequency of 10^−5^ to 10^−3^ and leads to false-positives during the negative selection [1]. Unlike true Janus replacement transformants (Km^s^Sm^r^), spontaneous revertants are Km^r^Sm^r^ since Janus is not replaced. To identify true allelic replacement, Sm^r^ clones resulted from the negative selection need to be duplicated on Km and Sm plates to screen for Km^s^ ones. When the transformation frequency is around 10^−7^, as reported for several pneumococcal strains [5], [6], more than 99% of the Sm^r^ clones would be false-positives, making the post-selection screening a time-consuming process.

To improve the efficiency of negative selection, we hypothesized that adding another counter-selectable marker, especially one not subject to gene conversion, to Janus would decrease the revertant frequency and reduce false-positives. Here we show that the *sacB* gene from *Bacillus subtilis* can be used in pneumococcus as an additional counter-selectable marker in Janus. The *sacB* gene encodes the enzyme levansucrase that converts sucrose to high molecular-weight fructose polymers (levans) [7], [8]. *sacB* is commonly used to mediate negative selection in gram-negative bacteria [9] because expression of *sacB* leads to cell death in the presence of sucrose (Su) [7]. In a few gram-positive bacteria, *sacB* was also shown to confer sucrose sensitivity (Su^s^) and function as a counter-selectable marker [10]–[12]. Because simultaneous reversion of both counter-selectable makers is much less likely, the addition of *sacB* in Janus reduces the revertant frequency to around 10^−9^ and greatly enhances negative selection efficiency.

## Materials and Methods

### Bacterial strains, DNA, and growth conditions

*S. pneumoniae* strains used in this study are described in Table 1. All strains were maintained in Todd-Hewitt broth supplemented with 0.5% yeast extract (THY) or on blood agar base no. 2 medium (Becton Dickinson, Sparks, MD) supplemented with 5% defibrinated sheep blood (Northeast Laboratory, Waterville, ME) (SBA). Antibiotic concentrations in SBA selective media were 500 mg/L for kanamycin and 200 mg/L for streptomycin. Genomic DNA was prepared by DNeasy Blood & Tissue Kit (Qiagen, Valencia, CA). Growth media and culture methods for genetic transformations have been described by Pozzi *et al*. [13]. The transformation of TIGR4 derivatives was induced by competence-stimulating peptide variant 2 (CSP-2) and the transformation of SpnYL002 was induced by CSP-1. The concentrations of donor DNA used in transformation steps were 200 ng/ml for chromosomal DNA and 100 ng/ml for PCR products.

**Table 1:** Bacterial strains and primers used in this study.

Strain or primer	Description	Source or reference
S. pneumoniae strains		
TIGR4S	Sm^r^ TIGR4 (serotype 4 isolate) by selection of mutations	[2]
TIGR4J	TIGR4S but *cps*::kan-*rpsL* ^+^, Km^r^Sm^s^Su^r^	[2]
SpnYL001	TIGR4S but *cps*::*sacB*-kan-*rpsL* ^+^, Km^r^Sm^s^Su^s^	This study
603	Serotype 6B clinical isolate, Km^s^Sm^s^	[14]
SpnYL002	Sm^r^ BR1014 (serotype 23B isolate) by selection of mutations	This study
SpnYL003	SpnYL002 but *cps*::kan-*rpsL* ^+^, Km^r^Sm^s^Su^r^	This study
SpnYL004	SpnYL002 but *cps*::*sacB*-kan-*rpsL* ^+^, Km^r^Sm^s^Su^s^	This study
Primers		
YL229	GATTGCGGCTATTTTTGGAA	This study
YL236	ATCCTTCCATTCATCCCCATAAGTGACC	This study
YL257	CCCTTATAAGCACGGGCACAAAAGTATGGCTAAAATGAGAATATCACCGGAATTG	This study
YL264	TATTATTTCCCTCCTCTTTTCTACAG	This study
YL265	CTGTAGAAAAGAGGAGGGAAATAATAAATGAACATCAAAAAGTTTGCAAAACAAGCAAC	This study
YL266	ACTTTTGTGCCCGTGCTTATAAGGG CGGCCATCGGCATTTTCTTTTGCG	This study

### Construction of the SJ cassette

The *sacB* coding region was amplified from the *SacB* plasmid (pCre-SacB-Zeo, kindly provided by Kenan Murphy of University of Massachusetts Medical School) by PCR using the primer pair YL265 and YL266. To construct a SJ cassette between DNA sequences corresponding to the genes that flank the capsular polysaccharide biosynthesis locus (*cps* locus), two DNA fragments, YL229-YL264 and YL257-YL236, were prepared by PCR using chromosomal DNA of TIGR4J as template. DNA fragment YL229-YL264 contains 1422-bp of the *cp*s upstream sequence (*dexB*) and 81-bp of the constitutive promoter sequence in Janus. DNA fragment YL257-YL236 contains Kan-*rpsL* ^+^ sequence of the Janus and 1363-bp of the *cps* downstream sequence (*aliA*). To assemble DNA fragments YL229-YL264, YL265-YL266 and YL257-YL236 into SJ, primers were designed to have 25-bp overlap at the junctions regions and the PCR products were ligated by using the Gibson Assembly Kit (New England BioLabs, Ipswich, MA) according to manufacturer's instructions. The assembly product was used as the template for PCR with primers YL229 and YL236. Product of the PCR was used to transform 403S and SpnYL002 with selection for resistance to kanamycin to create SpnYL001 and SpnYL004, respectively.

### Revertant frequency

Strains with the *cps* locus replaced by Janus (TIGR4J and SpnYL003) or SJ (SpnYL001 and SpnYL004) were grown to mid-log phase in THY and subjected to titer determination on SBA plates supplemented with Km (500 µg/ml), Sm (200 µg/ml), Su (10% w/v), or a mix of Sm and Su (Sm+Su, final concentrations 200 µg/ml and 10% w/v, respectively). For this and all subsequent titer determination steps, the result was denoted as one-half the detection limit when no colony was observed on a plate. Revertant frequency was calculated as the ratio of colonies growing on selection plates (Sm, Su, or Sm+Su) to the number growing on the Km plate.

### False-positive frequency and screen size

Genomic DNA from a serotype 6B strain (603) or no DNA control (None) was used to transform recipient strains TIGR4J and SpnYL001. Cultures of transformation were subjected to titer determination on SBA plates supplemented with Km, Sm, Su, or Sm+Su. The false-positive frequency was calculated as (T_S_/T_Km_)_None_/(T_S_/T_Km_)_603_, where T_S_ and T_Km_ are the concentrations of colony-forming units on a selection plate (Sm, Su, or Sm+Su) and on the Km plate, respectively. If the observed ratio was greater than 1 then the false-positive frequency was denoted as 0.99. To calculate the screen size (number of colonies on the selection plate to be examined after transformation) that would allow 95% probability of finding at least one true transformant (Km^s^) among revertants (Km^r^), the observed revertant frequency during transformation was used (3.34×10^−5^ for TIGR4J selection by Sm and 2.06×10^−9^ for SpnYL001 selection by Sm+Su). For a given transformation frequency, the screen size was calculated as maximum of (1, log(1–0.95)/log(1-f)), where f =  transformation frequency/(transformation frequency + revertant frequency).

## Results

**Figure 1:**
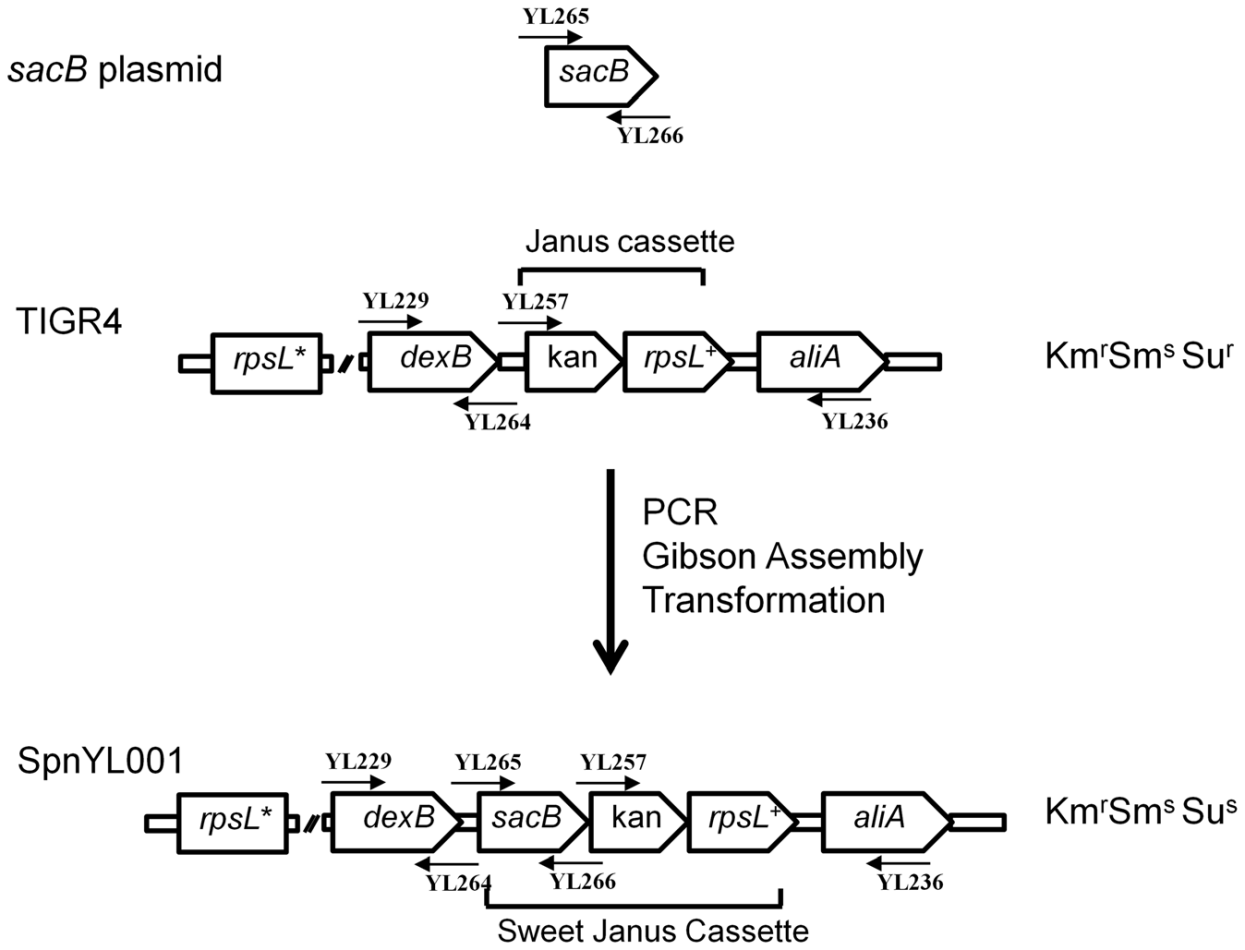
Construction of Sweet Janus (SJ) cassette in the *cps* locus of pneumococcus. Pentagons represent cassette elements and the *dexB* and *aliA* genes flanking the *cps* locus. Arrows represent oligonucleotides used to amplify fragments of the SJ cassette by PCR (see Table 1 for primer sequences). rpsL* represents the Sm^r^ mutant *rpsL* copy in the genomic region outside the Janus or SJ cassette. The PCR products were assembled into the SJ by using Gibson Assembly and the SJ was used to transform the TIGR4S strain to construct the SpnYL001 strain.

SJ was constructed by inserting the *sacB* coding sequence between the promoter region and the start codon of the Km resistance gene in the Janus cassette (Figure 1). The SJ was used to replace the *cps* loci in the TIGR4S and SpnYL002 strains to construct the SpnYL001 and SpnYL004 strains, respectively (Table 1). The SJ-carrying strains (Km^r^Sm^s^Su^s^) were subjected to titer determination on SBA plates supplemented with Km, Sm, Su, or a mix of Sm and Su (Sm+Su) to determine sensitivity to selective agents. Two Janus-carrying strains (Km^r^Sm^s^Su^r^), TIGR4J and SpnYL003, were also subjected to titer determination on the same set of SBA plates for comparison. Both SJ- and Janus-carrying strains showed spontaneous Sm^r^ revertant frequencies ranging from 10^−6^ to 10^−5^ (Table 2). For Janus-carrying strains, the Su^r^ titer was similar to the Km^r^ titer. In contrast, the Su^r^ titer was approximately 10^5^-fold lower than the Km^r^ titer for the SJ-carrying strains (Table 2). Moreover, growth of SJ-carrying strains on plates containing both Sm and Su (Sm+Su) was about 10^−9^-fold lower than the growth on Km plate (Table 2), which represented about 10^4^-fold additional growth reduction compared to when Sm or Su alone was used as selective agent. The results indicated that *SacB* in the SJ conferred Su^s^ in pneumococcus and functioned in synergy with *rpsL* ^+^ to reduce the spontaneous revertant frequency.

**Table 2:** Spontaneous reversion of SJ and Janus cassettes in two backgrounds

Strain	Growth (CFU/ml) with selection on				Revertant frequency		
	Km	Sm	Su	Sm+Su	Sm^r^	Su^r^	(Sm+Su)^r^
TIGR4J	1.8×10^9^	3.6×10^3^	1.6×10^9^	5.6×10^3^	2.0×10^−6^	NA	3.1×10^−6^
SpnYL001	6.3×10^8^	6.3×10^3^	9.9×10^3^	1#	1.0×10^−5^	1.6×10^−5^	1.6×10^−9^
SpnYL003	1.76×10^8^	6.36×10^3^	1.30×10^8^	5.76×10^3^	3.61×10^−5^	NA	3.27×10^−5^
SpnYL004	3.67×10^8^	7.88×10^3^	9.09×10^3^	1#	2.14×10^−5^	2.48×10^−5^	2.72×10^−9^

# Titer below the limit of detection was denoted as one-half the detection limit

NA: Revertant frequency not calculated because strains showed no Su sensitivity.

To test whether the low revertant frequency of SJ could enhance negative selection stringency, the TIGR4J and SpnYL001 strains were transformed with genomic DNA from a type 6B strain (603). The transformation cultures were subjected to titer determination on SBA plates with different selective agents (Table 3). Control transformations with no DNA added (None) were also performed. The false-positive frequency of negative selection was calculated as the ratio between the frequency of selected clones in the control transformation and frequency of selected clones in the DNA transformation. As shown in Table 3, when Sm alone was used as the selective agent, both Janus and SJ showed a high false-positive frequency close to 1. When Su alone was used as the selection agent, the false-positive frequency for SJ was also high (0.50, Table 3). However, when Sm and Su were used together (Sm+Su), the false-positive frequency for SJ decreased to 0.01 (Table 3). Further examination of 10 Sm^r^Su^r^ clones in the DNA transformation of SpnYL001 showed that all were Km^s^ and typed as serotype 6B. Thus, nearly all Sm^r^Su^r^ clones after DNA transformation of SpnYL001 resulted from replacement of SJ by the *cps* operon of the donor DNA. In fact, the frequency of the true transformants (Sm^r^Su^r^) was only 5.24×10^−7^, which was much lower than the spontaneous revertant frequency for either Sm or Su. The low transformation frequency could explain the high false-positive frequency observed when Sm or Su alone was used as the selective agent.

**Table 3:** Comparison of false positive frequency in the Janus- and SJ-mediated negative selection.

Recipient Strain	Donor DNA	Growth (CFU/ml) with selection on				False positive frequency		
		Km	Sm	Su	Sm+Su	Selection by Sm only	Selection by Su only	Selection by Sm+Su
TIGR4J	None	1.98×10^7^	6.61×10^2^	NA	NA	0.99	NA	NA
	603(Sm^S^)	2.28×10^7^	7.21×10^2^	NA	NA			
								
SpnYL001	None	4.85×10^7^	1.80×10^2^	2.70×10^2^	0.3#	0.99	0.50	0.01
	603(Sm^S^)	5.15×10^7^	1.83×10^2^	5.71×10^2^	27			

# Titer below the limit of detection was denoted as one-half the detection limit.

NA: No data was collected for the TIGR4J strain when Su was used in selection because TIGR4J showed no Su sensitivity.

**Figure 2:**
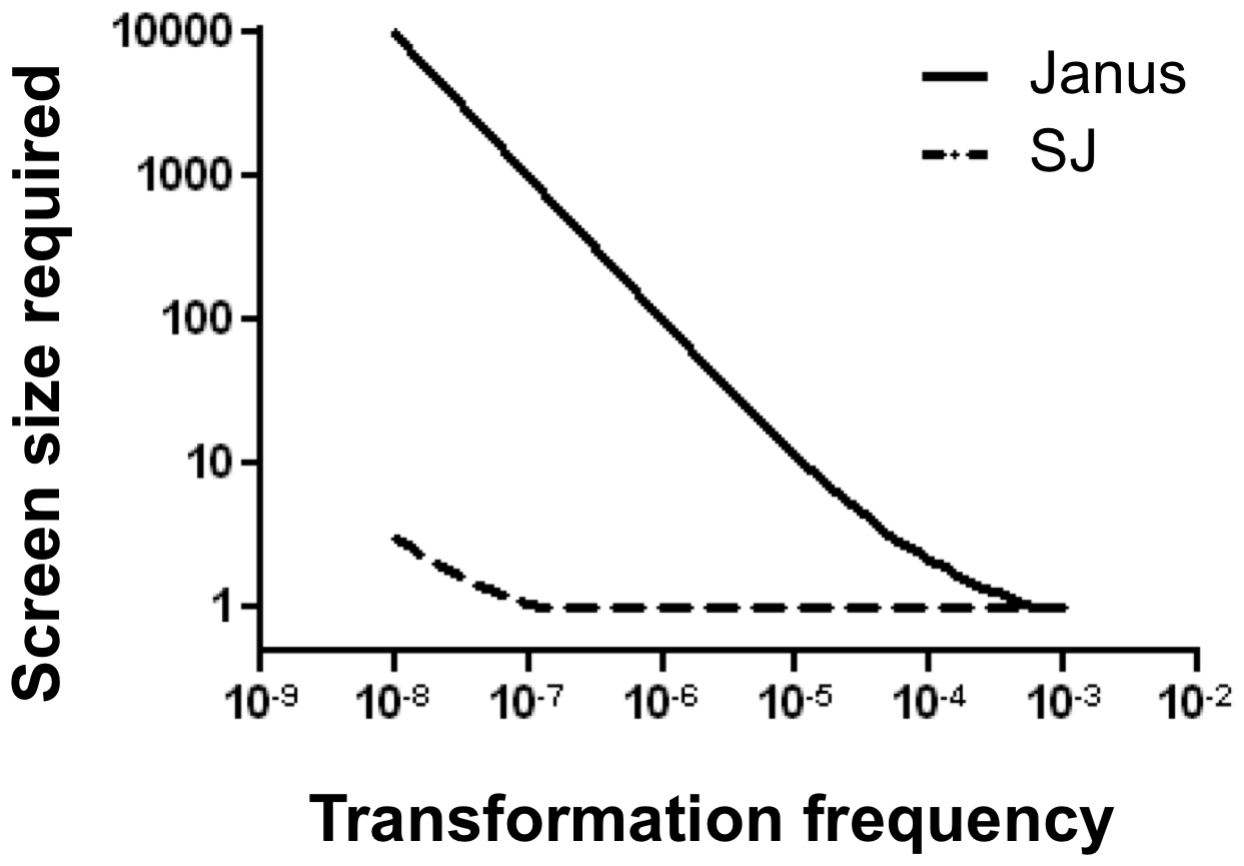
SJ allows more efficient identification of true transformants. Screen size (number of colonies on the selection plate to be examined after transformation) that would allow 95% probability of finding at least one true transformant (Km^s^) among revertants (Km^r^) at indicated transformation frequency was calculated for Janus (solid line) and SJ (dashed line). The observed revertant frequency during transformation of Janus and SJ (Table 3) was used in screen size calculation (see Materials and Methods for calculation details).

For both Janus- and SJ-mediated allelic exchange, an additional screening step is usually needed after the negative selection in order to identify true transformants (Km^s^) among spontaneous revertants (Km^r^). The screen size (number of clones to be examined) is determined by transformation frequency and spontaneous-revertant frequency. Using the observed revertant frequency in the presence of CSP-2, we estimated the screen size required to ensure 95% probability of finding at least one true transformant (Figure 2). The screen size required by SJ would be more than 100-fold lower than the screen size required by Janus when the transformation frequency is below 10^-6^ (Figure 2).

We observed that growing the SJ-carrying strains on a Su plate resulted in very small colonies. To test whether these colonies are true Su^r^ revertants, we duplicated 21 clones of SpnYL004 found on Su plate to another Su plate and a plate with no selective agent. 20 of these clones showed no growth on either plates, and 1 clone showed growth on both plates. For the one stable Su^r^ revertant of SpnYL004, we sequenced the *SacB* coding region and found a C to T substitution at position 1078, which will cause an arginine-to-cysteine change in the encoded protein.

## Discussion

In this study we constructed a modified Janus cassette, SJ, by adding an additional counter selectable marker, *sacB*, to Janus. Introduction of SJ to pneumococcus resulted in sensitivity to both Sm and Su. The frequency of SJ spontaneous double revertants (Sm^r^Su^r^) was about 10^4^-fold lower than the frequency of Janus spontaneous revertants (Sm^r^). Accordingly, the frequency of false-positives in the SJ-mediated negative selection was about 100-fold lower than what was seen for the Janus-mediated negative selection. We propose that the SJ cassette can greatly accelerate constructing marker-free allelic replacement or knockout in *Streptococcus pneumoniae* by reducing the amount of work required for post-selection screening.

The false-positive frequency during negative selection is determined by the relative frequency between transformation and spontaneous reversion since both events generate the selectable Sm^r^ phenotype. Transformation frequency was reported to vary among pneumococcal strains and a frequency below 10^−6^ was not unusual [5], [6]. Transformation conditions, including the choice of competence-stimulating peptide variants [13], can also influence transformation frequency. When transformation frequency is lower than 10^−6^ due to, for example, strain background or suboptimal transformation conditions, a Janus-mediated negative selection would require screening hundreds of Sm^r^ clones after selection (Figure 2) in order to identify a true transformant because vast majority of the Sm^r^ clones would be spontaneous revertants. In contrast, a screen size less than 10 would usually be sufficient for the SJ-mediate negative selection (Figure 2) due to its extremely low double revertant frequency.

Growth of the SJ-carrying strains, and not the Janus-carrying strains, was severely inhibited in the presence of sucrose (Table 2). The results suggested the *sacB* gene can confer sucrose-sensitivity, making it possible to use *sacB* alone as a counter-selectable marker in the Gram-positive pneumococcus. Compared to using *rpsL^+^*, using *sacB* alone to mediate negative selection in pneumococcus will have the advantage of not requiring a Sm^r^ background to perform allelic replacement, such that the strain after allelic replacement would not remain Sm^r^ and be truly maker-free. Use of *SacB* alone would work best when transformation frequency is high relative to the frequency of Su^r^ revertants; otherwise high false-positive frequency may result similar to the situation when *rpsL* ^+^ is used alone. In addition, the sucrose sensitivity resulting from expression of *sacB* has been shown in many Gram-negative bacteria but only in a few Gram-positive bacteria [9]. The basis of the toxicity is still unclear. It will be interesting to understand the mechanism by which *sacB* expression causes cell death of the Gram-positive pneumococcus in the presence of sucrose.

Growing the SJ-carrying strains on a Su plate resulted in very small colonies and most of these small colonies could not be cultured on either a Su plate or a plate with no selective agents. These observations suggested that many of the apparent Su^r^ revertants on the Su plate are phenotypic variants that did not acquire stable resistance to Su. It is possible that the small colony observed is composed of dead or dying bacteria accumulated during a slowing killing kinetics mediated by expression of the *SacB* gene in pneumococcus. Further investigation is needed to understand the source of the small colonies.
